# Assessment of Immunization Levels in Children with Chronic Diseases in a Tertiary Hospital

**DOI:** 10.3390/vaccines14080704

**Published:** 2026-08-14

**Authors:** Murat Ersoy, Betül Ulukol

**Affiliations:** 1 Child Health and Diseases Clinic, Mersin City Education and Research Hospital, Health Sciences University, Mersin 33240, Türkiye; 2 Division of Child Health and Diseases, Department of Internal Medicine, Faculty of Medicine, Ankara University, Ankara 06620, Türkiye; betul.ulukol@gmail.com

**Keywords:** vaccine, immunization, chronic disease, child

## Abstract

**Background/Objective:** Vaccination is one of the most effective and cost-effective preventive health measures for preventing infectious diseases. The literature reports that children with chronic illnesses have lower vaccination rates than healthy children and are at higher risk for preventable diseases. This study aimed to determine the immunization status of children with chronic illnesses, assess vaccination rates, and help raise awareness on this issue. **Material/Method:** Children aged 1 month to 18 years who presented to or were admitted to the Pediatric Clinic of Mersin City Education and Research Hospital between 1 March and 30 June 2024, and who were followed up for their chronic illness, and whose parents gave their consent, were included in the study. Sociodemographic data and information about routine and vaccines outside the national immunization schedule (Rotavirus, meningococcal, HPV, Influenza) were obtained from the parents. The status of vaccination of disease-specific recommended vaccines was also recorded. **Results:** A total of 256 children were included in the study (mean age 8.7 ± 4.69 years; 42.2% girls). A high rate of complete vaccination according to the routine vaccination schedule was observed. However, non-routine vaccinations were administered at low rates: rotavirus 9%, meningococcal 4.7%, and influenza 12.2%. The HPV vaccine was administered to only one patient in the study group. Recommended vaccines for the disease were administered to only 16.4% of eligible children. **Conclusions:** Children with chronic illnesses are at high risk for infection and complications. Increasing vaccine awareness among both healthcare professionals and parents will contribute to higher immunization rates. Comprehensive studies are needed to identify factors influencing vaccination in this population.

## 1. Introduction

The process of protecting a person against diseases through vaccination is called immunization [1]. Immunization, a global success story that saves countless lives every year, is divided into two categories: active and passive. Vaccines work together with our body’s natural defense mechanisms to reduce the risk of contracting a disease [2]. Vaccination programs in society have two purposes. First, to protect the vaccinated individual from the disease (individual immunity), and second, to ensure disease control in the community, which is called herd immunity. For these reasons, vaccination is a fundamental preventive health service [3]. Vaccination is one of the most effective and cost-effective preventive health practices in preventing infectious diseases [4]. Thanks to vaccination programs, the incidence of many infectious diseases has been significantly reduced, and some diseases have been almost completely eradicated. Nevertheless, approximately 1.7 million people worldwide die each year from vaccine-preventable diseases [5]. Vaccination programs have a preventive effect on morbidity and mortality in children, as well as reducing the incidence of infectious diseases [6]. The Expanded Immunization Program, initiated by the World Health Organization (WHO) in 1974, has played a significant role in increasing childhood vaccination rates. In our country, this program was implemented in 1981 and over the years the national vaccination schedule has been expanded and new vaccines have been included in the program [7].

Our 2024 vaccination schedule includes vaccines against Hepatitis B, diphtheria, pertussis, tetanus, pneumococcal disease, tuberculosis, haemophilus influenzae type B, poliomyelitis, Hepatitis A, measles, rubella, mumps, and chickenpox [8].

It is also recommended to administer vaccines for influenza, meningococcal, human papilloma virus (HPV) and rotavirus, which are not included in the standard vaccination schedule [9].

Chronic illness is defined as a health problem that requires continuous care, monitoring and medical intervention or that can restrict daily life [10]. According to the World Health Organization, chronic disease is defined as a persistent illnesses caused by irreversible pathological conditions that lead to permanent damage, require special training for the patient’s rehabilitation, and necessitate long-term monitoring and care [11]. According to 2023 data, 41 million people worldwide die each year from chronic diseases, accounting for 74% of all deaths globally [12]. In recent years, there has been a significant increase in the number of children with chronic illnesses [13]. Advances in medicine and new research are increasing survival rates for chronic and life-threatening childhood diseases. Recent literature shows a decrease in deaths among children with chronic diseases and that 90% of these children reach adulthood [14]. The literature also reports that children with chronic illnesses have lower vaccination rates than healthy children and are at higher risk when it comes to preventable diseases [15]. Bone marrow transplantation, organ transplantation, HIV, congenital and acquired immunodeficiencies, inflammatory diseases and immunomodulatory therapy use and chronic diseases such as asplenia/hyposplenia can increase risk for infections via suppression of the immune system or increase the likelihood of exposure to infectious agents both due to treatments and the diseases themselves [16]. Children with chronic diseases are a more susceptible group to infections. The disease itself, treatments affecting the immune system, frequent hospital visits and hospitalizations can increase the risk of infection in these children. In addition, infections that may develop can negatively affect the course of the chronic disease and lead to serious complications. Therefore, it is of great importance that immunization programs are implemented meticulously in children with chronic diseases [17].

The literature shows that vaccination rates in children with chronic diseases may be lower than in the general population [18,19]. Factors such as misperception of contraindications, disease activity and lack of information among families can cause delays or deficiencies in vaccination [20].

This study aimed to determine the immunization levels of children with chronic diseases, evaluate vaccination rates and contribute to raising awareness on this issue.

## 2. Materials and Methods

### 2.1. Study Design and Participants

This study was planned as a descriptive cross-sectional study. The research was conducted at the Pediatric Clinic of Mersin City Education and Research Hospital between 1 March 2024 and 30 June 2024. Children aged 1 month to 18 years who presented with a diagnosis of chronic disease were treated as outpatients or inpatients and followed up in subspecialty clinics were included and informed consent from patients’ parents was taken who approved to participate in the study. Patients’ parents who were not signed informed consent form and whose vaccination status data were missing were excluded from the study.

Our study was planned to be conducted with at least 224 patients according to power analysis. A total of 350 chronically ill children were asked to participate in the study and final 256 patients agreed.

### 2.2. Data Collection

Data was collected from the parents of the patients participating in the study using a face-to-face survey method. Since digital records became available in 2015, standardization has been achieved based on clinical data; medical conditions and vaccination history were obtained using a standardized data form during on-site parent interviews.

The survey form basically includes the following information:

- The child’s age, gender, and educational status.
- Chronic disease diagnosis and the medical specialty in which it is followed.
- Duration of illness and hospital admission history.
- Medications used.
- Parents’ age, education level, and income level.
- Questions about the status of administration of 2024 Ministry of Health routine vaccination schedule and self-paid vaccines.

The 2024 Ministry of Health routine vaccination schedule includes 3 doses of the Hepatitis B vaccine (HepB) at birth and at 1 and 6 months of age, 1 dose of the Bacillus Calmette–Guérin vaccine (BCG) at 2 months, 4 doses of the combined vaccine diphtheria, tetanus, and acellular pertussis, inactive poliomyelitis vaccine, haemophilus influenzae type b (DaPT-İPV, Hib) at 2, 4, 6, and 18 months of age; conjugated pneumococcal vaccine (PCV) at 2, 4, and 12 months of age, oral poliomyelitis vaccine (OPV) at 6–18 months of age; single-dose chickenpox at 12 months, measles, mumps, and rubella combined live vaccine (MMR) at 12 and 48 months of age, and again at 48 months of age; combined vaccine diphtheria, tetanus, and acellular pertussis, inactive poliomyelitis vaccine (DaPT-İPV) without Hib; two doses Hepatitis A at 18–24 months; diphtheroid tetanus at age 13 [8]. Self-paid vaccines are rotavirus, meningococcus, HPV and influenza vaccines recommended outside of the routine schedule.

Those who received all the vaccinations required for their age according to the Ministry of Health’s vaccination schedule were considered fully vaccinated. Data from non-routine self-paid vaccines were evaluated separately.

Since the Hepatitis A vaccine was included in the routine vaccination schedule in 2012, children under and over 12 years of age have been evaluated separately. Also, the evaluation of the varicella-zoster vaccine, which was added to the routine vaccination schedule in 2013, was based on an 11-year-old age group.

### 2.3. Statistical Analysis

Statistical analyses were performed using IBM SPSS Statistics for MacOS, version 29 (IBM Corp., Armonk, NY, USA). Categorical variables were expressed as numbers and percentages [*n* (%)] while continuous variables were expressed as mean ± standard deviation and median values. Comparisons between categorical variables were performed using the Pearson chi-square test or Fisher’s exact test, as appropriate. All statistical tests were two-sided, and a *p*-value < 0.05 was considered statistically significant.

### 2.4. Ethical Statement

Ethical approval for this study was obtained from the Toros University Scientific Research and Publication Ethics Committee with decision number 23.02.2024/39. Furthermore, the necessary permissions were obtained from the Mersin City Education and Research Hospital Education Planning Commission before the study was planned. At the beginning of the study, all participants’ parents or legal guardians were contacted and provided with detailed information about the purpose of the study, the process, participants’ rights and privacy policies. An informed consent form was prepared and signed. The study was conducted in accordance with the ethical standards specified in the Helsinki Declaration and its subsequent amendments.

## 3. Results

The average age of the children was 8.7 ± 4.7 years, and the median age was 8 years. A total of 42.2% of the children were girls and 57.8% were boys. The percentage of preschool-aged was 39.5%, the percentage attending kindergarten was 4.7%, and the percentage of children receiving primary and secondary education was 55.8% (Table 1). The most frequently followed specialties for children were endocrinology (30.9%), neurology (24.2%), and pulmonary diseases (15.6%).

It was found that approximately 40.2% of them had incomes at or below the minimum wage. The ages of the mothers ranged from 22 to 55, with an average age of 36. A total of 11.7% of the mothers were illiterate and 29.3% were high school graduates or higher. A total of 10.5% of the mothers were employed, while 89.5% were housewives. The ages of the fathers ranged from 26 to 65, with an average age of 38. A total of 3.5% of the fathers were illiterate, and 40.6% were high school graduates or higher. A total of 96.9% of the fathers were employed, while 3.1% were unemployed (Table 1).

According to the routine schedule, fully vaccinated patient rates are high. The full vaccination rate according to age was found to be 98.4% for Hepatitis B and BCG vaccines; 97.3% for DTaP-IPV-Hib vaccine; 96.1% for Conjugate Pneumococcal Vaccine (PCV); 96.4% for Measles–Mumps–Rubella (MMR) vaccine; 98.1% for DTaP-IPV vaccine; 94% for Oral Polio Vaccine (OPV); and 88% for Adult-onset Diphtheria (Td) (Table 2). Due to the inclusion of the Hepatitis A vaccine in the routine vaccination schedule in 2012, children under and over 12 years of age were evaluated separately. Of the 239 children evaluated, the vaccination rate was found to be 92.5% for children under 12 and 84.6% for children over 12. The evaluation of the varicella vaccine, which was added to the routine vaccination schedule in 2013, was conducted based on an age group of 11 years. When evaluated by age group, 96.6% of the 117 children under 11 years old and 93.4% of the 76 children over 11 years old received the varicella vaccine.

Hence, rates are low for non-routine vaccines. Rotavirus vaccine was administered to 9% of children, meningococcal ACWY to 9.7%, meningococcal B to 4.6%, influenza to 12.2%, and HPV to only one child. Recommended vaccines for the disease are recommended for all children, with an application rate of 16.4%; of these, 73.8% are influenza and 33.3% are polysaccharide pneumococcal vaccine; 6% are meningococcal vaccine and 60% are palivizumab vaccine (Table 3).

It was determined that 30.9% *(n* = 79) of the children were diagnosed in endocrinology, 24.2% (*n* = 62) in neurology, 15.6% (*n* = 40) in pulmonary diseases, 7.4% (*n* = 19) in metabolism, 6.3% *(n* = 16) in genetics and rheumatology, 5.1% (*n* = 13) in nephrology, 3.5% (*n* = 9) in gastroenterology, oncology and cardiology, 3.1% (*n* = 8) in dermatology, and 0.4% (*n* = 1) in infectious diseases and immunology.

When evaluated according to the type of chronic disease, the rate of complete administration of routine vaccinations is ≥90% in children with neurological disease, while the rate of non-routine vaccinations is low (rotavirus 12.9%, meningococcal ACWY 6.4%, meningococcal B 3.2%, and influenza 6.5%). Similarly, in children with endocrine disease, routine vaccinations are above 90%, and non-routine vaccinations are below 10% (Table 4).

The effects of parental age, education and income level on children’s vaccination status were examined. For most vaccines, no statistically significant difference was found between the age group of mothers or fathers and full vaccination status (*p* > 0.05). The complete vaccination rate for the Measles–Mumps–Rubella (MMR) vaccine was found to be higher in mothers under the age of 36 and fathers under the age of 38. This difference was not statistically significant (*p* = 0.9, *p* = 0.925) (Table 5).

Full vaccination rates for all vaccines, except MMR and Hepatitis A, showed similar results across all education levels of mothers. Full vaccination rates for both MMR and Hepatitis A increased with increasing education level. No statistically significant difference was found (*p* = 0.964 for MMR, *p* = 0.081 for Hepatitis A) (Table 5).

Regarding fathers’ education levels, the complete vaccination rate for all vaccines except MMR and Hepatitis A showed similar results across all education levels. The complete vaccination rate for MMR and Hepatitis A increased with increasing education level. This was particularly statistically significant for MMR (*p* < 0.001) (Table 5).

Full vaccination rates for both routine and non-routine vaccines were similar across all family income levels.

## 4. Discussion

Vaccination is critically important for protecting children, especially those with chronic illnesses, from infectious diseases. Our study evaluated vaccination rates in children with chronic illnesses and revealed two important findings. First, the coverage rates of vaccines included in the national vaccination program are generally high. Second, the rate of use of non-routine vaccines recommended in this vulnerable population is quite low. This is consistent with the trend of undervaccination reported previously in the literature [17,20]. The high coverage rate observed for routine vaccines is consistent with the national vaccination policies in Turkey, which provide widespread access to childhood vaccines [21].

In contrast, coverage rates for non-routine vaccines such as rotavirus, meningococcal, HPV and influenza are significantly lower. Similar trends have been reported in previous studies from different countries, suggesting that the use of vaccines not included in national vaccination programs tends to remain limited. When evaluated on a country basis, meningococcal vaccination rates were found to be 5.7% in Spain [21], 67.1% in İtaly [22] and 28% in the Netherlands [23]. Meningococcal vaccine administration rate was found to be 4.7% in our study which indicates that the risk of infection remains high. Similarly, the influenza vaccination rate (12.1%) was significantly lower than the rates reported previously in the study by Diallo et al. at the University of Lille [24] Alauzet et al., which involved 402 patients, ranging from 15% to 46.5% [25]. Several factors may have contributed to this, including a lack of awareness among parents, inadequate physician advice, and misconceptions about vaccine safety in children with chronic illness. Particularly noteworthy in our study was the extremely low HPV vaccination rate; only one patient received the vaccine. This finding reflects the limited integration of the HPV vaccine into national immunization strategies in many countries and underlines the need for increased awareness and policy interventions. In the study conducted by Çelik and İncesoy-Özdemir in our country, it was determined that the HPV vaccination rate was 0.2%; rotavirus vaccination rate was 7.6%, followed by meningococcal vaccination rate at 3.1%. The age groups of the children and the presence of chronic diseases were not specified in the study [26]. Previous studies from Türkiye have also reported suboptimal uptake of vaccines not included in the National Immunization Program among healthy children, suggesting that barriers to optional vaccination may extend beyond the chronic disease population [27].

When analyzed according to disease, routine vaccination rates were high in children with neurological and endocrine diseases, while non-routine vaccination rates were low. These results indicate that vaccination of risk groups in children with chronic diseases is not adequately provided, and this is reflected in the literature. This is supported by similar findings in the studies of Gallone et al. and Cerutti et al. [28,29].

Despite the high PCV vaccination ratio, the rate of the pneumococcal polysaccharide vaccine (PPSV23), which is recommended especially for high-risk groups, was found to be insufficient (33.3%). Although this finding is higher than the rate previously reported by Gallone et al. (7.2%) [28], it suggests that a significant proportion of high-risk children is inadequately protected against invasive pneumococcal disease. This gap highlights a critical failure in risk-based vaccination strategies.

Parental factors also play a role in vaccination coverage. While parental age was not significantly associated with overall vaccination status, MMR vaccination rates were higher among younger parents. Higher parental education levels were associated with better adoption of MMR and Hepatitis A vaccines, suggesting that health literacy may influence vaccination decisions. These findings are consistent with previous studies showing the impact of parental education on immunization coverage [30]. In contrast, income level did not appear to significantly affect vaccination rates, which may support the idea that access to free vaccination services can mitigate socioeconomic inequalities [31].

The consistently low vaccination rates in high-risk groups underscore the need for targeted interventions. Raising awareness among both healthcare professionals and caregivers is essential. Studies have shown that increased awareness of disease severity and vaccine-preventable complications significantly increases vaccination rates, particularly for influenza and pneumococcal vaccines [14]. Furthermore, it is recommended that comprehensive studies are conducted to examine other factors affecting the vaccination status of children with chronic diseases. Also, our study evaluated vaccination coverage but did not assess vaccine immunogenicity or antibody responses. Further studies incorporating serologic evaluations and clinical outcomes are needed to determine whether vaccination gaps are accompanied by reduced immune protection in children with chronic diseases.

The most important strength of this study is its comprehensive assessment of multiple chronic disease groups and its provision of a holistic perspective on vaccination coverage. However, the study also has limitations, such as its single-center design, the failure to examine factors affecting vaccination status and a lack of generalizability. Moreover, unavailability of the exact dates of vaccine administration is one of the limitations of this study. Therefore, although age-appropriate vaccination status could be assessed, vaccination timeliness and the potential influence of disease duration or hospitalization history on delays in vaccination could not be evaluated.

## 5. Conclusions

A multidisciplinary approach plays a crucial role in the monitoring of children with chronic illnesses. Taking precautions against the illness itself or the conditions that may arise during the treatment process will eliminate negative outcomes. Our study showed that while most routine vaccinations were completed, non-routine vaccination rates were low. This indicates that the risk of infection persists in children with chronic illnesses and that strategies to increase awareness among families and healthcare professionals need to be developed. The correlation between parental education level and the vaccination rates of some vaccines underlines the importance of information and education interventions. There is a need for comprehensive studies examining factors affecting vaccination status, including the level of knowledge of families regarding the disease and vaccination, and similarly, the attitudes and knowledge of physicians regarding the immunization of children with chronic illnesses. We believe that future studies of this kind could lead to an increase in vaccination rates among patients in the at risk group.

## Figures and Tables

**Table 1:** Demographic data of children and families.

Variables	*n* (%)	Variables	*n* (%)
Children’s age		Mother’s Education	
0–4 years	59 (23.0)	Illiterate	30 (11.7)
5–9 years	81 (31.6)	Primary-secondary	151 (59.0)
10–17 years	116 (45.3)	*High* School and above	75 (29.3)
Children Gender		Mother’s Working Status	
Female	108 (42.2)	Housewife	229 (89.5)
Male	148 (57.8)	Working	27 (10.5)
Educational Status of Children		Father’s education	
Preschool	101 (39.5)	Illiterate	9 (3.5)
Kindergarten	12 (4.7)	Primary-secondary	143 (55.8)
Primary School	55 (21.5)	High School and above	104 (40.6)
Secondary School	51 (19.9)	Father’s Working Status	
High School	37 (14.5)	Not Working	8 (3.1)
		Working	248 (96.9)

**Table 2:** Full vaccination rate of routine vaccines.

Routine Vaccination	%
Hepatitis B	98.4%
BCG	98.4%
DTaP-IPV-Hib	97.3%
Conjugate Pneumococcal Vaccine (PCV)	96.1%
MMR	96.4%
DTaP-IPV	98.1%
Oral Polio Vaccine (OPV)	94%
Td	88%
Hepatitis A	<12 age 92.5, >12 age 84.6
Varicella	<11 age 96.6, >12 age 93.4

**Table 3:** Distribution of Recommended Vaccinations for the Disease.

Variables (*n* = 256)	*n* (%)
Vaccines recommended for the disease	
İnfluenza (N:256) *	31 (12.2)
Polysaccharide Pneumococcal Vaccine (N:18) **	6 (33.3)
Meningococcal Vaccine (N:33) ***	2 (6)
Palivizumab (N:5) ****	3 (60)

* Recommended for all chronic patients. ** Number of patients for whom the polysaccharide pneumococcal vaccine should be recommended. *** Number of patients for whom meningococcal vaccine should be particularly recommended. **** Number of patients for whom palivizumab should be recommended.

**Table 4:** Vaccination status according to the specialty.

Specialty	HepB	BCG	DTaP-İPV-Hib	PCV	MMR	DTaP	OPV	Td	HepA	Varicella	Rotavirus	MenACWY	Men-B	HPV	Influenza
Neurology (*n* = 62)	61 (98.4)	59 (95.2)	60 (96.8)	58 (93.5)	58 (93.5)	42 (97.6)	55 (90.2)	10 (76.9)	48 (87.2)	59 (95,2)	8 (12.9)	4 (6.4)	2 (3.2)	0	4 (6.5)
Endocrinology (*n* = 79)	79 (100)	79 (100)	78 (98.7)	77 (97.4)	79 (100)	68 (98.5)	74 (93.6)	31 (93.9)	68 (89.4)	76 (96.2)	6 (7.5)	4 (5.0)	1 (1.3)	0	4 (5.0)
Chest Diseases (*n* = 40)	40 (100)	40 (100)	39 (97.5)	40 (100)	39 (97.5)	38 (100)	38 (97.4)	10 (90.9)	38 (97.4)	39 (97.5)	6 (15.0)	4 (10.0)	2 (5.0)	0	0
Gastroenterology (*n* = 8)	8 (100)	8 (100)	8 (100)	8 (100)	8 (100)	7 (100)	8 (100)	4 (100)	7 (100)	8 (100)	0	1 (12.5)	1 (12.5)	0	0
Genetics (*n* = 13)	11 (84.6)	11 (84.6)	11 (84.6)	11 (84.6)	11 (84.6)	6 (100)	10 (90.9)	1 (100)	11 (91.6)	13 (100)	0	1 (7.6)	0	0	1 (7.6)
Metabolism (*n* = 19)	19 (100)	19 (100)	19 (100)	18 (94.7)	17 (100)	13 (92.8)	17 (94.4)	4 (66.7)	13 (81.2)	16 (94.1)	0	0	0	0	2 (10.5)
Rheumatology (*n* = 13)	13 (100)	13 (100)	13 (100)	13 (100)	11 (84.6)	13 (100)	12 (92.3)	5 (100)	12 (92.3)	12 (92.3)	2 (15.4)	1 (7.7)	1 (7.7)	0	1 (7.6)
Nephrology (*n* = 9)	9 (100)	9 (100)	9 (100)	9 (100)	8 (100)	7 (100)	8 (100)	4 (100)	7 (87.5)	7 (87.5)	1 (11.1)	1 (11.1)	1 (11.1)	0	1 (11.1)
Oncology (*n* = 8)	8 (100)	8 (100)	8 (100)	8 (100)	7 (87.5)	8 (100)	8 (100)	2 (66.7)	8 (100)	8 (100)	0	0	0	0	0
Hematology (*n* = 16)	16 (100)	16 (100)	16 (100)	16 (100)	16 (100)	14 (100)	16 (100)	6 (100)	16 (100)	16 (100)	0	1 (6.2)	1 (6.2)	0	1 (6.2)
Cardiology (*n* = 8)	8 (100)	8 (100)	8 (100)	8 (100)	8 (100)	3 (100)	8 (100)	1 (100)	8 (100)	8 (100)	0	1 (12.5)	0	0	3 (37.5)

**Table 5:** Parent’s age and educational status for MMR and Hepatitis A vaccines.

	Mother’s Educational Status					*p*-Value
	Illiterate	Primary School	Secondary School	High School	University	
MMR	28 (11.5)	68 (27.9)	76 (31.1)	52 (21.3)	20 (8.2)	0.964
Hepatitis A	23 (10.6)	57 (26.4)	70 (32.4)	49 (22.7)	17 (7.9)	0.081
	Father’s educational status					*p*-value
	Illiterate	Primary school	Secondary school	High school	University	
MMR	7 (2.9)	59 (24.2)	76 (31.1)	73 (29.9)	29 (11.9)	<0.001
Hepatitis A	7 (3.2)	48 (22.2)	68 (31.5)	67 (31.0)	26 (12.0)	0.168
	Vaccination rate according to mother’s and father’s age for MMR					
	Mother’s age ≤ 36	Mother’s age > 36	*p*-value	Father’s age ≤ 38	Father’s age > 38	*p*-value
MMR	128 (96.2)	118 (95.9)	0.9	121 (96)	123 (96.9)	0.925

## Data Availability

The datasets used and/or analyzed in this study are available from the corresponding author upon reasonable request.

## Abbreviations

HPV	Human Papilloma Virus
WHO	World Health Organization
PCV	Pneumococcal Conjugate Vaccine
PPSV23	Pneumococcal Polysaccharide Vaccine
MMR	Measles, Mumps, and Rubella
HepB	Hepatitis B
BCG	Bacillus Calmette–Guérin
DaPT	Diphtheria, tetanus, and acellular pertussis
İPV	Inactive Poliomyelitis Vaccine
Hib	Haemophilus Influenzae Type b
OPV	Oral Poliomyelitis Vaccine
